# Pansharpening Model of Transferable Remote Sensing Images Based on Feature Fusion and Attention Modules

**DOI:** 10.3390/s23063275

**Published:** 2023-03-20

**Authors:** Hui Liu, Liangfeng Deng, Yibo Dou, Xiwu Zhong, Yurong Qian

**Affiliations:** 1 School of Information Science and Engineering, Xinjiang University, Urumqi 830014, China; liuhui@Stu.xju.edu.cn; 2 Key Laboratory of Software Engineering, Xinjiang University, Urumqi 830008, China; 3 Key Laboratory of Signal Detection and Processing, Xinjiang Uygur Autonomous Region, Urumqi 830046, China; 4 School of Software, Xinjiang University, Urumqi 830008, China

**Keywords:** panchromatic sharpening, attention mechanism, convolutional neural network, hyperspectral image sharpening

## Abstract

The purpose of the panchromatic sharpening of remote sensing images is to generate high-resolution multispectral images through software technology without increasing economic expenditure. The specific method is to fuse the spatial information of a high-resolution panchromatic image and the spectral information of a low-resolution multispectral image. This work proposes a novel model for generating high-quality multispectral images. This model uses the feature domain of the convolution neural network to fuse multispectral and panchromatic images so that the fused images can generate new features so that the final fused features can restore clear images. Because of the unique feature extraction ability of convolution neural networks, we use the core idea of convolution neural networks to extract global features. To extract the complementary features of the input image at a deeper level, we first designed two subnetworks with the same structure but different weights, and then used single-channel attention to optimize the fused features to improve the final fusion performance. We select the public data set widely used in this field to verify the validity of the model. The experimental results on the GaoFen-2 and SPOT6 data sets show that this method has a better effect in fusing multi-spectral and panchromatic images. Compared with the classical and the latest methods in this field, our model fusion obtained panchromatic sharpened images from both quantitative and qualitative analysis has achieved better results. In addition, to verify the transferability and generalization of our proposed model, we directly apply it to multispectral image sharpening, such as hyperspectral image sharpening. Experiments and tests have been carried out on Pavia Center and Botswana public hyperspectral data sets, and the results show that the model has also achieved good performance in hyperspectral data sets.

## 1. Introduction

In recent years, with the emergence of many high-resolution Earth observation satellites, such as “GeoEye1”, “SPOT6”, and “GaoFen-2”, remote sensing applications have been widely used in research fields such as geography and land surveying. In these research fields, remote sensing images are often required to have high spectral resolution and high spatial resolution. However, as far as the design of the current remote sensing system is concerned, the spectral and spatial resolution often cannot be maintained at a high level at the same time. The images acquired by different sensors are different in terms of geometric features, spectral resolution, and spatial resolution. Some sensors acquire rich scene spectral information but lack sufficient spatial information, such as multi-spectral images (MS). On the other hand, some sensors are good at capturing spatial information, but cannot capture reliable spectral information, such as panchromatic images (PAN). Images with high spatial resolution provide subtle geometric features, while images with high spectral resolution provide rich spectral information, which can be used to identify and analyze targets. To make full use of the information provided by multi-spectral images and panchromatic images, the usual method is to fuse low-resolution multi-spectral images with high-resolution panchromatic images of the same scene to generate images with more detailed spatial and spectral structures, that is, pansharpening.

Remote sensing images need to be preprocessed. One of the most basic preprocessing methods is the panchromatic sharpening of remote sensing images, which plays a vital role in subsequent tasks such as target detection, classification, and the semantic segmentation of remote sensing images [1,2,3,4]. In early research and development, panchromatic sharpening can be broadly classified into the following four categories: (1) multiresolution analysis (MRA) [5,6,7,8,9,10,11]; (2) component substitution (CS) [12,13,14,15,16,17,18,19]; (3 ) mixed methods (combining CS and MRA) [20,21,22]; and (4) model-based methods [23,24,25,26,27]. Among the above four algorithms, the component substitution method has the characteristics of high space and high fidelity, and this method is straightforward to implement. However, the component replacement method also has some disadvantages, such as ignoring the local differences between MS and PAN images, resulting in obvious spectral distortions in the final image fusion stage. Secondly, in terms of preserving spectral information, although the MRA method has achieved good results, the effect of image fusion is significantly affected by the number of image decomposition and filter types. There are also strict requirements for image registration. Based on the hybrid method, although the high spatial fidelity of CS and the ability of MRA to preserve spectral information are combined, this is not enough, and the fused image still has the problems of spectral distortion and spatial structure distortion. The model-based method does an excellent job of solving the spectral distortion problem, but the solution process of the inversion model is time-consuming and complicated.

In recent years, in the field of computer vision, convolutional neural networks (CNNs) have been widely used and have achieved excellent results. Therefore, for the problem of panchromatic sharpening, many remote sensing researchers have attempted to use deep learning methods to solve it. For example, Masi et al. [28] applied a convolution neural network to panchromatic sharpening and made a major research breakthrough. Based on the single-image super-resolution reconstruction algorithm [29], a three-layer convolutional neural network (PNN) was proposed, which regards panchromatic sharpening as image super-resolution reconstruction. A panchromatic sharpening network based on domain knowledge was designed by Yang et al. [30]. In order to deal with the panchromatic sharpening problem, Liu et al. [31] designed a two-branch fusion network.

In order to obtain a better fusion effect, methods based on deep learning can take advantage of the slight spectral distortion and the feature extraction ability of the robust convolutional neural network. In existing methods, either neural networks are used to extract spatial details, or the panchromatic sharpening problem is treated as a super-resolution reconstruction problem. However, artifacts or spectral distortion still exist in the fusion results produced by these methods. This is because previous methods often assume that panchromatic and multispectral images contain different information. There needs to be a better solution for defining spatial and spectral information and how to extract these two types of information separately. In addition, the defining characteristic of a PAN image is that it contains less spectral information but more spatial information. The defining characteristic of an MS image is that it contains more spectral information but has a lower spatial resolution. Therefore, how to design a model that can combine the advantages of the two types of images to generate an applicable hyperspectral panchromatic image is an urgent problem to be solved. However, the existing deep learning methods usually only consist of the linear stitching of simple feature maps in feature fusion, and not all feature maps play a positive role in the final fusion process.

To overcome these problems, this article proposes a dual-branch fusion net-work based on the attention mechanism to optimize feature fusion and solve the problem of pansharpening. In addition, we applied the proposed model to hyperspectral image sharpening (HSI sharpening), and the experimental results show that the model also achieved better results on hyperspectral datasets. This study makes the following contributions:

1. We provide an end-to-end deep learning model for remote sensing image panchromatic sharpening and realize the reconstruction in the CNN feature domain to generate higher quality panchromatic sharpened images;
2. In the feature fusion module, a channel attention mechanism is introduced to optimize feature fusion, allowing the network to focus on crucial information;
3. Our network is a general model that can be directly applied to the hyperspectral image (HSI) sharpening and panchromatic image sharpening. For example, our experiments show that our method can achieve state-of-the-art performance both qualitatively and quantitatively.

The organization of the rest of this paper is as follows: In the second part, related works and the materials needed for this article are introduced. The third part elaborates on the pansharpening model of remote sensing images based on the attention optimization feature fusion proposed in this paper. The fourth part provides the experimental details, including the experimental settings, comparison experiments, and ablation experiments. In the fifth part, the proposed model is applied to HSI sharpening to prove the universality of the proposed model. Finally, this article is summarized.

## 2. Related Work

### 2.1. Pansharpening Based on Deep Learning

In recent years, the application of convolution neural networks to remote sensing images has attracted increasing attention from researchers. For example, the design of a high-performance panchromatic sharpening algorithm represents one such study, using the characteristics of remote sensing images to perform spatiotemporal fusion and spatial spectrum fusion without increasing the costs relating to equipment and manpower. Yuan [32] and others improved PNN by adding a multi-scale feature extraction module, which could make full use of the spatial features in high-resolution images and achieve an improved fusion effect. Ma et al. [33] proposed an unsupervised generation countermeasure network (GAN), which could better preserve the spatial features in PAN images. Liu et al. [34] proposed a GAN network for the panchromatic sharpening of remote sensing images and the effective fusion of MS and PAN images. On the basis of the literature [30], Fu et al. [35] proposed a grouping multi-scale gap network structure to expand the perception domain of each network layer, effectively obtain fine-grained multi-scale context features, and improve the quality of fused images. Zhou et al. [36] proposed an unsupervised pansharpening network based on perceptual loss and an automatic encoder. Liu et al. [37] based on the fusion results of different adaptive ground averaging methods, combined with the complementary properties of CS and MRA methods, proposed a generalized sharpening weighted network. Li et al. [38] proposed a multi-scale perceptual dense coding convolutional neural network to generate high-quality pansharpened images. The authors in [39] proposed a model-based deep sharpening method called the gradient projection-based pansharpening neural network (GPPNN). This model regards panchromatic images and low-resolution multispectral images as two optimization problems related to depth prior to regularization and uses a gradient projection algorithm to solve these two problems. The edge information part-guided convolutional sparse coding network, SCSC-PNN, proposed by Xu et al. [40] is applied to panchromatic sharpening in the field of remote sensing. This model mainly uses edge information regularization to segment low-resolution multispectral images and obtain a panchromatic image correlation feature map and a panchromatic image non-correlation feature map. HARNN [41] is a residual neural network based on the mixed attention mechanism. The encoder attention module is developed in the feature extraction part to solve the accuracy calculation of ground object recognition caused by spectral distortion and a lack of spatial detail in the pansharpening method. Yan et al. [42] proposed a model-driven and data-driven network, which combines model-driven and data-driven methods, and introduces a depth prior as its implicit regularization, thus improving its data adaptability and representative ability to be applied to multimodal fusion tasks. Guan et al. [43] proposed a hyperspectral pansharpening method of a multi-level double-attention guided fusion network (MDA-Net). This method uses a three-stream structure to enable the network to solve the hyperspectral pansharpening problem by combining the inherent characteristics of each input and their correlation at the same time.

Although the above methods have made some achievements, their spectral and spatial information have not been fully used for MS and PAN image fusion, respectively; that is to say, the lack of valuable information fusion leads to a certain spectral distortion and blur in the final fusion image. In addition, when performing feature fusion, only simple linear stitching is performed on the feature map, which usually does not meet the requirements of the pansharpening task. Therefore, in order to overcome the existing problems, this paper proposes an attention-based two-branch fusion network to optimize feature fusion.

### 2.2. Attention Mechanism

Attention mechanisms have been widely used in several research areas, such as natural language processing and computer vision. They enable the network to focus on relevant information and filter unnecessary information like a human. The attention mechanism is divided into the channel, spatial, and hybrid domains. The purpose of the attention mechanism is to better adjust the feature learning process by assigning different weights to different positions.

SENet [44] is an early visual model used to explore the channel attention mechanism. Through the “squeeze-incentive” process, different weights are obtained for each feature channel, different weights are assigned to different channels, and the channels are added to the attention mechanism. Zhang et al. [45] introduced SENet into the field of super-resolution reconstruction and constructed very deep residual channel attention networks (RCAN), which achieved better super resolution (SR) performance. The convolutional block attention module (CBAM) [46] is used to focus the two latitudes of the network space and the channel at the same time through the series space and channel attention modules, in order to help the network to understand the “what” and the “where” aspects of the attention. The attention mechanism improves the feature representation ability of neural networks by focusing more on critical features while reducing the attention paid to other features. Li et al. [47] proposed a new way of using the attention mechanism, using channel attention to merge the characteristics of the two branches. Furthermore, Dai et al. [48] proposed a multi-scale attention feature fusion module to replace the traditional summation and splicing methods for completing feature fusion.

## 3. Methods

**Figure 1:**
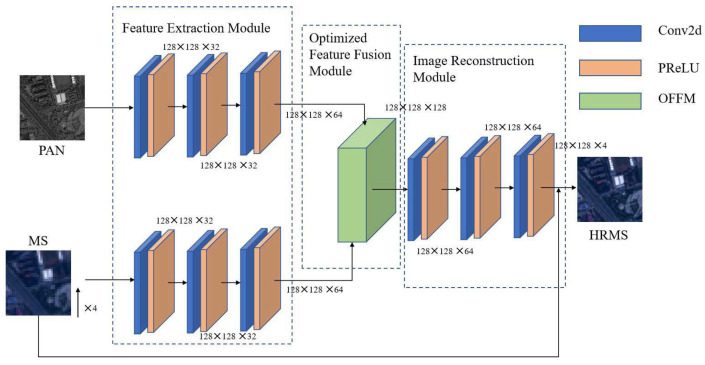
The overall framework diagram of the dual-branch remote sensing image fusion network based on the attention mechanism to optimize feature fusion. The inputs are MS and PAN images, and the output is HRMS. The network architecture is mainly composed of the following three sub-modules: (1) feature extraction module; (2) optimized feature fusion module (OFFM); and (3) image reconstruction module.

The structure of the converged network proposed in this paper is shown in Figure 1. It consists of three modules: feature extraction, optimized feature fusion, and image reconstruction. This section first introduces the specific content of these three modules, and then introduces the loss function used.

### 3.1. Feature Extraction Module

The feature extraction module in this paper uses two sub-networks with the same structure but different weights. As shown, the upper subnetwork takes a single-band panchromatic (PAN) image as input, and the lower subnetwork takes a four-band multispectral (MS) image as input. Both feature extraction subnetworks consist of three consecutive convolution kernels as a 3 × 3 convolutional layer, followed by a parametric rectified linear unit (PReLU). Most CNN architectures use the average or max pooling to obtain rotation- and scale-invariant features, but the pooling operation is not used in the fusion network described in this article because in pansharpening, detailed information is more critical.

### 3.2. Optimized Feature Fusion Module

The complementary information of the panchromatic image and the multispectral image is extracted using two feature maps obtained by the feature extraction module. For panchromatic sharpening, we fuse the extracted feature maps to obtain a multispectral image with high spatial and spectral resolution.

**Figure 2:**
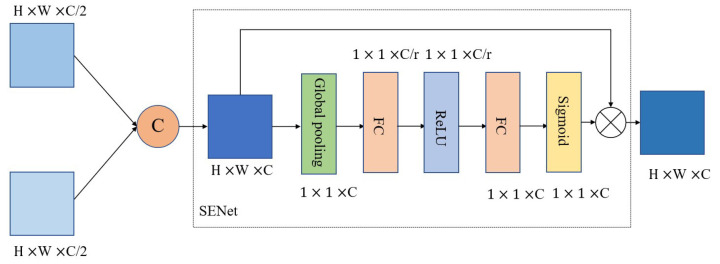
Network structure of the optimized feature fusion module.

The importance of the feature map to be fused with the fusion result varies, as does the amount of information carried. Therefore, valuable information needs to be enhanced, and useless information needs to be suppressed, meaning each channel must be weighted. Therefore, we first linearly concatenate the characteristic graphs of the two sets of complementary information, and then, through a channel attention mechanism SENet [44], we obtain a one-dimensional vector containing the number of channels of the characteristic graph, which represents the importance weight of each channel, and then apply the weight to the corresponding channels, so as to enhance the useful information and suppress the role of useless information. The network structure diagram of the optimized feature fusion module (OFFM) is shown in Figure 2.

### 3.3. Image Reconstruction Module

After feature fusion, we must recover high-resolution multispectral images from the fused features. Here, we use the convolution of the three-layer 3 × 3 convolution kernels to reconstruct the fused feature images and recover the HRMS images of the four bands. At the same time, we also use a long jump connection to directly transfer the input MS image, which has been upsampled to the same size as the PAN image by spectral mapping, to the output, which complements the spectral information of the reconstructed image. This point will also be proved in the ablation experiment, demonstrating the influence of this long jump connection on the fusion result.

### 3.4. Loss Function

The loss function is another key factor affecting the image quality of super-resolution reconstruction, besides the network structure. The ℓ2 loss function is often used in image reconstruction tasks [28,29,30]. ℓ2 loss can smooth the image and punish larger outliers while being less sensitive to smaller outliers. For further improvement, because the ℓ1 loss function can handle other small outliers and preserve the edge information of the image, we use the ℓ1 loss function to train the network. The ℓ1 loss function can be expressed as Equation (1). In the ablation experiment, we also prove through experiments that the ℓ1 paradigm can obtain better fusion results than the ℓ2 paradigm.

(1) $$\ell 1=\frac{1}{N}\sum_{i=1}^{N}|h\left(X_{p}^{\left(i\right)},X_{M}^{\left(i\right)},\theta \right)-Y^{\left(i\right)}{|}_{1},$$

where *N* is the number of small batch training samples, $X_{p}^{\left(i\right)}$ and $X_{M}^{\left(i\right)}$ are PAN images and low-resolution MS images, $Y^{\left(i\right)}$ is the corresponding high-resolution MS image, and θ is the parameter of the fusion network.

### 3.5. Evaluation Indicators

In order to compare the proposed method with some previous methods, we used six widely used metrics to evaluate them quantitatively.

(1) The peak signal-to-noise ratio (PSNR) [49] is defined as
  (2) $$PSNR=10\cdot \log_{10}\left(\frac{MAX_{I}^{2}}{MSE}\right)=20\cdot \log_{10}\left(\frac{MAX_{I}}{\sqrt{MSE}}\right)$$
  where MAXI is the maximum value that represents the color of the image point. The higher the PSNR value between two images, the less distorted the reconstructed image relative to the high-resolution image. MSE is defined as
  (3) $$MSE=\frac{1}{mn}\sum_{i=0}^{m-1}\sum_{j=0}^{n-1}{\left\|I\left(i,j\right)-K\left(i,j\right)\right\|}^{2}$$
  where I and K are two images of size m × n, one of which is the noise approximation of the other.
(2) The structural similarity (SSIM) index
  SSIM [50] measures the overall fusion quality by calculating the mean, variance, and covariance of the fused image and the reference image. The SSIM measurement consists of three contrast modules, namely, brightness, contrast, and structure. Given two images, X and Y, of size M × N, the means and variances of X and Y and the covariance are represented by $u_{x}$, $u_{y}$, $\delta_{x}^{2}$, $\delta_{y}^{2}$, and $\delta_{xy}$, respectively. The comparison functions that define the brightness, contrast, and structure are
  (4) $$\iota \left(X,Y\right)=\frac{2\mu_{x}\mu_{y}+c_{1}}{\mu_{x}^{2}+\mu_{y}^{2}+c_{1}}$$
  (5) $$c\left(X,Y\right)=\frac{2\delta_{x}\delta_{y}+c_{2}}{\delta_{x}^{2}+\delta_{y}^{2}+c_{2}}$$
  (6) $$s\left(X,Y\right)=\frac{\delta_{xy}+c_{3}}{\mu_{x}\mu_{y}+c_{3}}$$
  The combination of these three component factors is the SSIM indicator, which is defined as
  (7) $$SSIM\left(X,Y\right)={\left[l(X,Y)\right]}^{\alpha }{\left[c(X,Y)\right]}^{\beta }{\left[s(X,Y)\right]}^{\gamma }$$
  The closer the SSIM value is to 1, the higher the similarity between the two images.
(3) Spectral angle mapper (SAM)
  SAM [51] calculates the angle between the HRMS image and the fusion result to evaluate the spectral quality of the fusion result. The smaller the value, the better the spectral quality, with an ideal value of 0.
  (8) $$SAM=\arccos\left(\frac{\left(I_{\alpha }J_{\alpha }\right)}{\|I_{\alpha }\left\|\right\|J_{\alpha }\|}\right)$$
  where $I_{a}$ and $J_{a}$ are the pixel vectors of the fused image and the reference image, respectively, at the distance point α.
(4) Relative dimensionless global error in synthesis (ERGAS)
  ERGAS [52] can more comprehensively reflect the quality of the fusion result. The smaller the value of ERGAS, the better the result, with an ideal value of 0.
  (9) $$ERGAS=100\frac{h}{l}\sqrt{\frac{\sum_{i=1}^{N}\left(RMSE^{2}\left(B_{i}\right)/M_{i}^{2}\right)}{N}}$$
  where *h* is the resolution of the high-resolution image, *l* is the resolution of the low-resolution image, *N* is the number of bands, $B_{i}$ is the MS image, and $M_{i}$ is the average of the emissivity value of the MS image.
(5) Spatial correlation coefficient (SCC)
  SCC [53] is used to evaluate the similarity of the spatial details of the fused image and the reference image, using a high-pass filter to extract the high-frequency information of the reference image, and to calculate the correlation coefficient (CC) [54]. This article uses a high Laplacian filter to obtain a high frequency, as follows:
  (10) $$F=\left[\begin{array}{ccc} -1 & -1 & -1\\ -1 & 8 & -1\\ -1 & -1 & -1 \end{array}\right]$$
  A higher SCC means that most of the spatial information of the PAN image is injected during the fusion process. The SCC is calculated between the fused image and the reference image. The final SCC is averaged over all bands of the MS image. The CC is calculated as
  (11) $$CC=\frac{\sum_{i=1}^{w}\sum_{j=1}^{h}\left(X_{i,j}-\mu_{X}\right)\left(Y_{i,j}-\mu_{Y}\right)}{\sqrt{\sum_{i=1}^{w}\sum_{j=1}^{h}{\left(X_{i,j}-\mu_{X}\right)}^{2}{\left(Y_{i,j}-\mu_{Y}\right)}^{2}}}$$
  where *X* is the fused image, *Y* is the reference image, *w* and h are the width and height of the image, respectively, and μ represents the average value of the image.
(6) Quality index (Q)
  *Q* [55] combines three factors to calculate image distortion: correlation loss, brightness distortion, and contrast distortion. It is defined as
  (12) $$Q=\frac{|\sigma_{Z_{1},Z_{2}}|}{\sigma_{Z_{1}}\cdot \sigma_{Z_{2}}}\cdot \frac{2\sigma_{Z_{1}}\cdot \sigma_{Z_{2}}}{\sigma_{Z_{1}}^{2}+\sigma_{Z_{2}}^{2}}\cdot \frac{2|\bar{Z_{1}}|\cdot |\bar{Z_{2}}|}{|\bar{Z_{1}}{|}^{2}\cdot |\bar{Z_{2}}{|}^{2}}$$
  where $Z_{1}$ and $Z_{2}$ represent the b-th band of the fused image and the reference image, respectively. When *Q* is 1, this represents the best fidelity for reference.

## 4. Experiment

### 4.1. Dataset Introduction

We trained and tested our network on two datasets collected by GaoFen-2 and SPOT6, and compared it with a variety of advanced methods. Gaofen-2 is the first civilian optical remote sensing satellite independently developed by China, with a spatial resolution better than 1 m. It was launched on 19 August 2014. It has two cameras with high resolution (1 m panchromatic and 4 m multispectral). Launched on 9 September 2012, the SPOT6 satellite collects multispectral images with a spatial resolution of 6 m and full-color images with a spatial resolution of 1.5 m, including red, green, blue, and near-infrared. The relevant information on the GaoFen-2 and SPOT6 satellites is shown in Table 1.

### 4.2. Experimental Setup

**Figure 3:**
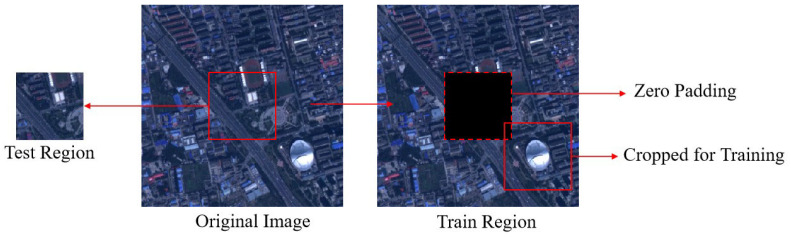
Examples of MS image training and testing regions for the GaoFen-2 dataset.

We trained and tested our network on the GaoFen-2 and SPOT6 datasets, respectively. We cropped the sub-regions of 32 × 32 and 128 × 128 in the center from the MS and PAN image pairs as test images and used the remaining regions for training. Specifically, in each iteration of training, we randomly cut out 32 × 32 and 128 × 128 image pairs with the same spatial resolution from the training area for use as training images. Our training area and test area did not overlap, which was achieved by filling the test area with zeros in the training phase. Figure 3 shows the MS image of GaoFen-2 as an example to introduce the division of the training area and test area.

Our goal was to generate a multispectral image with the same size and spatial resolution as the PAN image. We evaluated the proposed model by comparing the obtained results with nonexistent reference images. According to the Wald, protocol [55], we first preprocessed the image with a 5 × 5 Gaussian filter with a standard deviation of 2. Using the raw MS image (HRMS) as a reference, four downsampled PAN and blurred low-resolution MS images were used as input. In the network, in order to make the input MS image match the resolution of the PAN image, the MS image was upsampled using the bicubic interpolation method. Additionally, our network was implemented using PyTorch, using the Adam optimizer to minimize the loss. The training was performed on an Nvidia 3090 GPU, and the learning rate was set to 0.0004. A total of 30,000 epochs were trained.

### 4.3. Ablation Experiment

**Figure 4:**
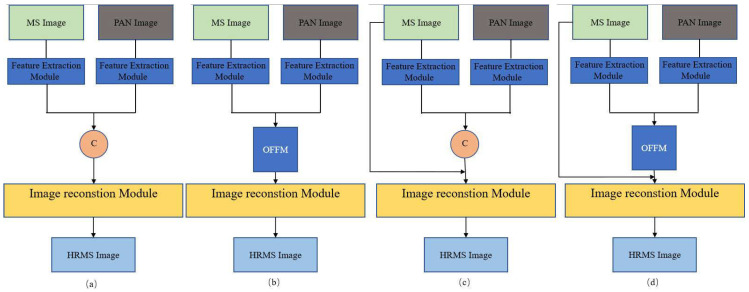
Ablation experiment. (**a**) Original. (**b**) Attention-original. (**c**) Skip-original. (**d**) Our-ℓ2.

To further verify the effect of the attention mechanism, spectral mapping, and the selection of the loss function on the impact of our proposed model, taking the Gaofen-2 dataset as an example, we first conducted ablation experiments on the following four models (as shown in Figure 4) with ℓ2 as the loss function, and then replaced the loss function with our proposed ℓ1 loss function, verifying that this loss function can improve the obtained results.

1. The original feature extraction and image reconstruction network (Original);
2. Using our attention-based optimization feature fusion module on the original network (Attention-original);
3. The addition of spectral mapping to the original network (Skip-original);
4. Using our optimized feature to fuse fuzzy and spectral mapping on the original network (Our-ℓ2).

The quantitative indicators of the experimental results of these four models on the GaoFen-2 dataset are shown in Table 2. From Table 2, we can see that when the long-skip connection of spectral mapping is added separately, both SAM and ERGAS are improved. This shows that such spectral mapping is beneficial to the maintenance of the fusion result. At the same time, we can also find that the PSNR, SSIM, SCC, and Q indicators improved to a certain extent. This is because as the network deepens, the features obtained by convolution are often more advanced, and these more high-level features map the semantic and abstract information of the image. Therefore, recovering the detailed texture of an image is difficult for high-level features. Such a long-hop connection directly transfers the low-level features of the input to the output, which also solves this problem to a certain extent. From Table 2, we can also see that when using our optimized feature fusion module alone, PSNR, SSIM, SCC, and Q are all improved. This shows that optimizing feature fusion is effective in space preservation and that assigning different weights to fusion features is more conducive to pansharpening tasks.

In order to combine the advantages of the two, we used spectral mapping and optimized feature fusion, simultaneously, as shown in Figure 4d. It can be seen from Table 2 that the results of Our-ℓ2, both in terms of the spectral index and the spatial index, are significantly higher than those of the original, demonstrating that our-ℓ2 model can save spectrum and space.

**Figure 5:**
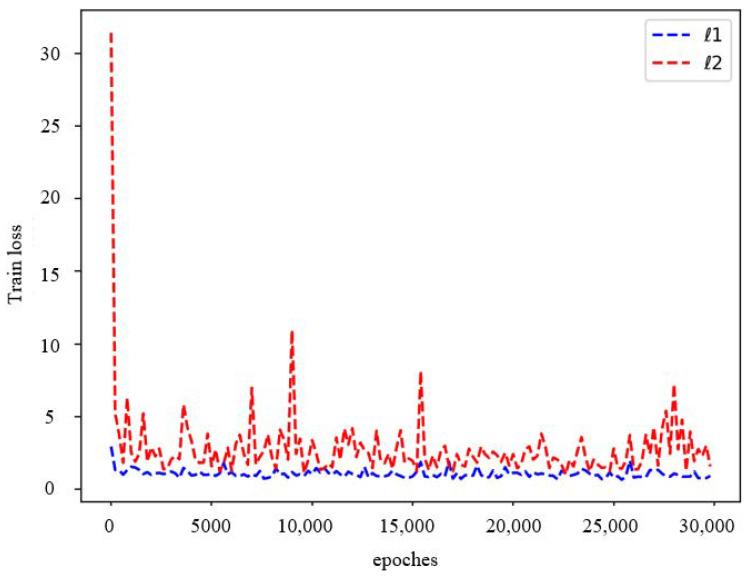
Changes in the loss function when using ℓ1 and ℓ2 in the training process.

In order to verify that ℓ1 is conducive to improving the performance of the model, we use the model with ℓ2 and ℓ1 as the loss function. The change in the loss function during the training process is shown in Figure 5. We can find that the ℓ1 loss function can reduce the training error and improve the network convergence. Therefore, we use the ℓ1 loss function to train our network. From the last row of Table 2, we can also see that when using ℓ1 as the loss function, all indicators achieved the best results, which indicates that the ℓ1 loss function is more conducive to pansharpening.

### 4.4. Comparison with Other Algorithms

There are currently several widely used techniques, and in this section, we compare the proposed method with these techniques, including principal component analysis (PCA) [56], the intensity-based saturation (IHS) transform fusion method [57], MTF-GLP with high-pass modulation (MGH) [58], deep-learning-based PNN [28], PanNet [30], TFNet [31], ResTFNet [31], and the multi-scale deep convolutional neural network (MSDCNN) [32].

**Figure 6:**
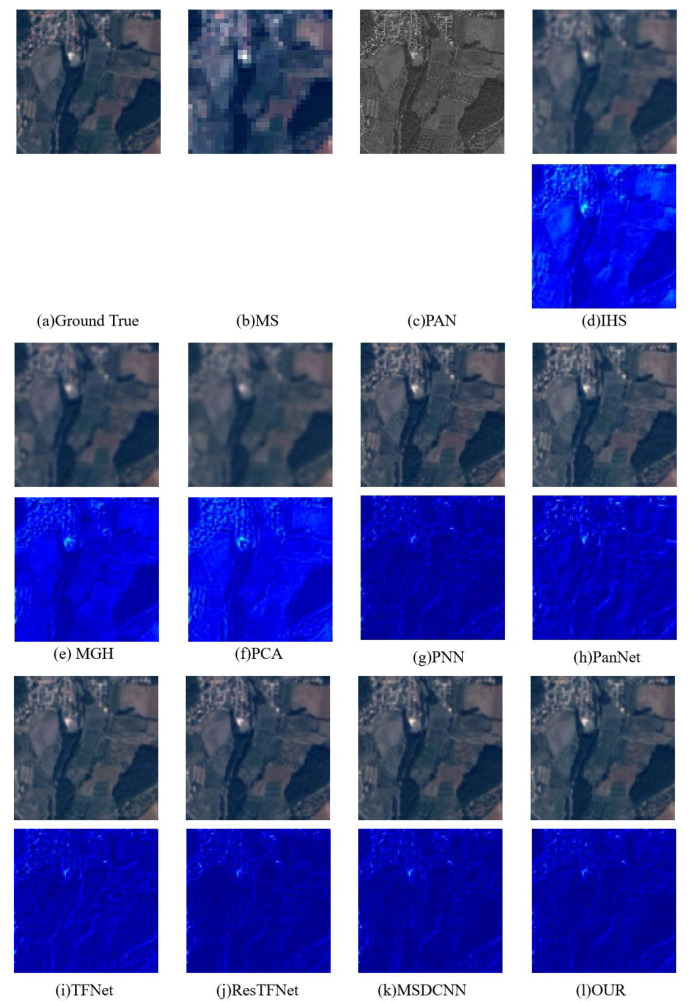
Pansharpening results on the SPOT6 satellite dataset. The first line is the fusion result of the SPOT6 image, and the second line is the image obtained by pseudo-coloring the differences between the fusion result of the first line and the ground truth RGB image (that is, (**a**)). All images are displayed in true color (red, green, and blue being the three bands).

**Figure 7:**
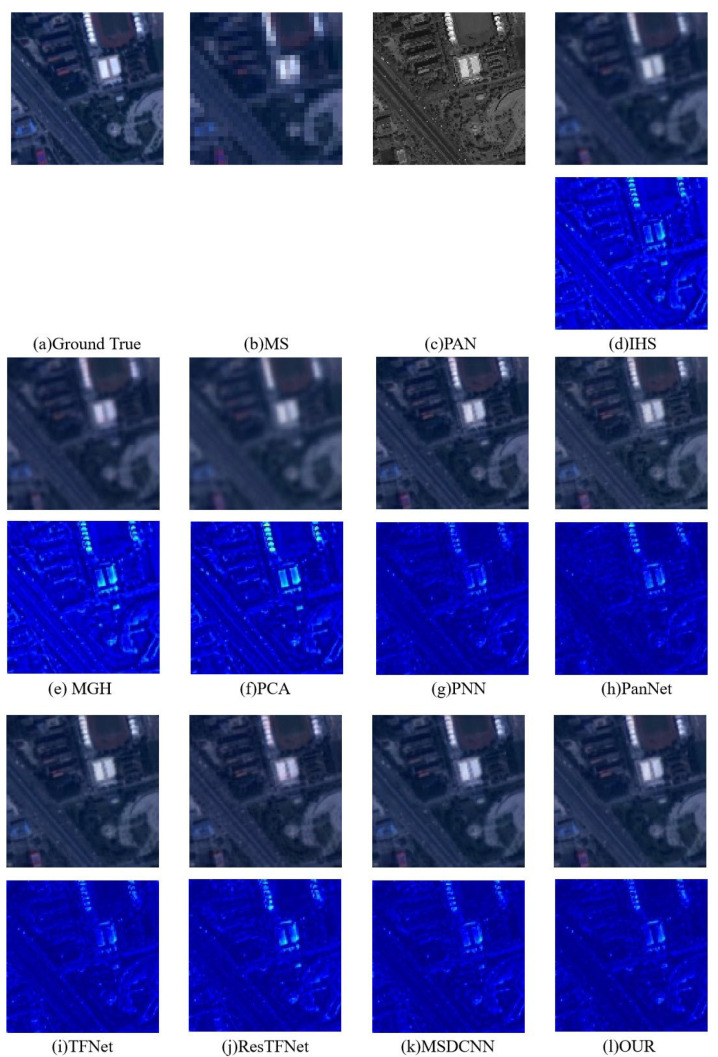
Pansharpening results on the GaoFen-2 satellite dataset. The first line is the fusion result of the GaoFen-2 image, and the second line is the image obtained by pseudo-coloring the differences between the fusion result of the first line and the ground truth RGB image (that is, (**a**)). All images are displayed in true color (red, green, and blue being the three bands).

We conducted a qualitative analysis of the simulated dataset. Figure 6 and Figure 7 show the pansharpening results of different algorithms on the two datasets. From the fusion results in Figure 6, we can see that IHS, MGH, and PCA have spectral distortions and some obvious spatial information loss, with PCA having the most obvious spatial and spectral distortions. Several other methods based on deep learning can produce visually satisfactory pansharpened images, but PanNet and TFNet have a certain loss and blurring of spatial details. Images obtained by our method can preserve both spectral information and relatively more detailed spatial information. From the fusion results on the GaoFen-2 dataset, it can be seen that PCA, MGH, and HIS also experience a severe loss of spectral and spatial information, especially PCA. Deep learning methods can achieve better fusion results, but RestFNet and PanNet have some details missing.

To more accurately evaluate the spatial and spectral distortion, we also highlight the difference between the fused image and the ground truth, that is, the residual map. The residual map in the second row shows that our model has relatively fewer details and textures, indicating that it is the best in terms of space preservation. At the same time, the overall smooth area of our model is shown in dark blue, indicating that all differences are close to 0, while other residual maps more or less contain obvious areas. Figure 6d–i and Figure 7d–f, in particular, show that the error is large. This shows that our method achieves better spectral retention.

At the same time, we also compare the algorithms from a quantitative perspective. Table 3 and Table 4 show the quantitative indicators on the two satellite datasets. The results in Table 3 and Table 4 show that although our model is not dominant in terms of model size, it has the best results for both spatial and spectral indicators and is also suboptimal in time. The results show that our model has the best performance in terms of spectrum preservation and spatial reconstruction. At the same time, MSDCNN, based on the deep learning method, also achieved better fusion performance, surpassing traditional methods in both spectral and spatial indicators. On the GaoFen-2 dataset, TFNet achieved great performance, and on the SPOT6 dataset, PNN and ResTFNet also achieved great results. The results show that deep learning is still a very effective method at present, showing great potential in solving problems related to pansharpening. Then, we added the quantitative analysis of time and model parameter quantity. From the data in the two data sets in Table 3 and Table 4, we can see that the model we proposed is only inferior to the PNN model and superior to other traditional models and models based on deep learning. We analyze that the reason why our model does not reach the optimal value in time and model parameters is that the double-branch structure increases the computational cost.

## 5. Hyperspectral Image Sharpening

Our model is directly applicable to other types of multispectral image sharpening models, and the proof is given below. In this section, the proposed model is applied to hyperspectral image (HSI) sharpening. In remote sensing image processing, HIS sharpening has attracted more and more attention. It was designed to fuse high-resolution MS images and low-resolution hyperspectral images in order to obtain images with high spatial and spectral resolution.

We compare the proposed model with the seven latest models of TF-Net [31], Res-TFNet [31], MSD-CNN [32], SSF-CNN [59], Con-SSFCNN [59], and SSR-NET [60] on the Pavia Center (Pavia) and Botswana hyperspectral datasets. Table 5 and Table 6 show the experimental results on the Pavia dataset and the Botswana dataset. It can be seen that our model has the best performance in all indicators. From the results, it can be seen that our network has the best performance in terms of preserving spatial and spectral information under the effect of HIS sharpening. This also proves that our network can be applied to different tasks and that it is a general model.

**Figure 8:**
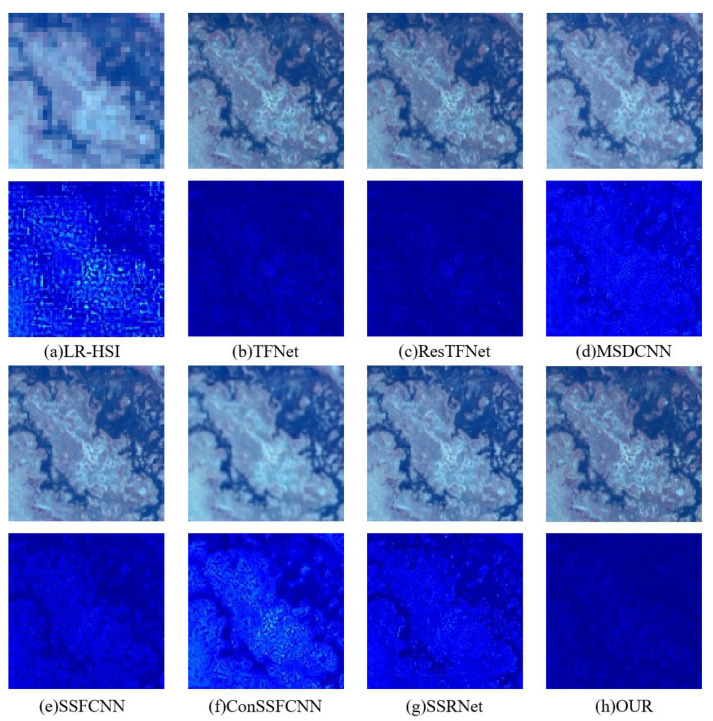
Sharpening results on the Botswana dataset. The first row is the fusion result, and the second row is an image obtained by pseudo-coloring the differences between the fusion result of the first row and the ground truth RGB image. All images are displayed in true color (red, green, and blue being the three bands).

**Figure 9:**
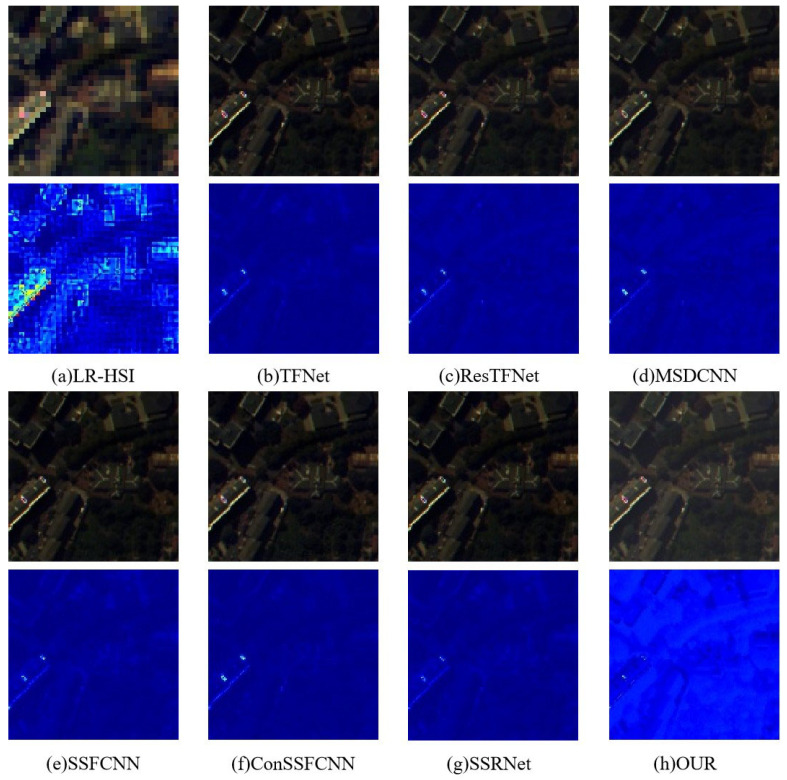
Sharpening results on the Pavia dataset. The first row is the fusion result, and the second row is an image obtained by pseudo-coloring the differences between the fusion result of the first row and the ground truth RGB image. All images are displayed in true color (red, green, and blue being the three bands).

At the same time, in Figure 8 and Figure 9, we also give the sharpening results of our model and other models on the Pavia and Botswana datasets for visual comparison. We can see that the images generated by TFNet, ResTFNet, MSDCNN, SSFCNN, ConSSFCNN, and SSRNet are clearly blurred. On the contrary, our model can maintain space and spectrum at the same time. We use pseudo colors in the corresponding residual maps to reflect the sharp differences between the results and the ground truth. We can see that the residual map of our model is displayed in dark blue as a whole, indicating that all of the differences are close to 0. Other residual maps contain more or less obvious areas, indicating that the error is larger, which also indicates that our method achieves better spectral preservation.

## 6. Conclusions

In this article, we propose a dual-branch fusion network based on attention-optimized feature fusion for the panchromatic sharpening of remote sensing images in the feature domain. This network is an end-to-end model that only requires the input of panchromatic and multispectral images to generate high-resolution multispectral images. Complementary information is extracted from the input image through two sub-networks with the same structure and different weights. The feature fusion is optimized through a channel attention mechanism to consider the relationship between other channels of the fusion features so that the network focuses more on critical information to improve the retention performance of the fused image in terms of spatial and spectral information. Compared with existing algorithms, this method shows better performance.

## Figures and Tables

**Table 1:** Spectral and spatial features of multispectral and panchromatic images from the SPOT6 and GF-2 satellites.

Satellite	Spectral Wavelength (nm)					Spatial Resolution (m)	
	PAN	Blue	Green	Red	Nir	PAN	MS
GaoFen-2	450–900	450–520	520–590	630–690	770–890	0.8	3.2
SPOT6	455–745	455–525	530–590	625–695	760–890	1.5	6

**Table 2:** The results of ablation experiments on the GaoFen-2 dataset (bold indicates the best results).

Module	OFFM	Long Skip	ℓ2	ℓ1	PSNR	SSIM	SAM	ERGAS	SCC	Q
Original			*√*		29.7071	0.8414	0.0715	3.5173	0.9561	0.7686
Attention-original	*√*		*√*		29.8053	0.8490	0.0695	3.4657	0.9571	0.7799
Skip-original		*√*	*√*		29.8339	0.8530	0.0670	3.4826	0.9572	0.7879
Our-ℓ2	*√*	*√*	*√*		29.9908	0.8566	0.0659	3.4099	0.9587	0.7957
Our	*√*	*√*		*√*	**30.1202**	**0.8659**	**0.0626**	**3.3609**	**0.9601**	**0.8092**

**Table 3:** Quantitative evaluation on the SPOT6 dataset (bold indicates the best results).

Methods	PSNR	SSIM	SAM	ERGAS	SCC	Q	TIME(MS)	MODLESIZE(M)
PCA	19.2991	0.5359	0.3617	10.9139	0.7619	0.4612	-	-
IHS	23.3756	0.5845	0.1063	7.565	0.7669	0.4933	-	-
MGH	22.8081	0.6123	0.0988	8.154	0.798	0.5431	-	-
PNN	28.7644	0.848	0.0762	3.0857	0.9159	0.749	**21.24**	0.31
PanNet	28.2992	0.8289	0.0802	3.361	0.9022	0.7115	34.17	**0.08**
TFNet	28.7404	0.8461	0.0785	3.1123	0.9146	0.7473	79.19	9.03
ResTFNet	28.627	0.8497	0.0775	3.0764	0.9142	0.7554	103.53	8.56
MSDCNN	28.8715	0.8509	0.0754	3.0556	0.9187	0.7545	98.49	1.01
Our	**29.4593**	**0.8721**	**0.0702**	**2.7903**	**0.9321**	**0.7892**	22.8	8.93

**Table 4:** Quantitative evaluation on the GaoFen-2 dataset (bold indicates the best results).

Methods	PSNR	SSIM	SAM	ERGAS	SCC	Q	TIME(MS)	MODLESIZE(M)
PCA	20.4468	0.3736	0.2375	13.305	0.7212	0.3063	-	-
IHS	21.1052	0.3786	0.1521	12.3192	0.7242	0.3188	-	-
MGH	21.1358	0.436	0.1723	12.2693	0.7892	0.3929	-	-
PNN	29.4323	0.8321	0.0721	3.6438	0.9524	0.7609	58.24	0.31
PanNet	29.0734	0.8192	0.0752	3.7975	0.9477	0.7444	**23.43**	**0.08**
TFNet	29.4638	0.8382	0.0723	3.6067	0.9527	0.7694	89.97	9.03
ResTFNet	28.6892	0.8162	0.0761	3.9423	0.9437	0.7411	84.92	8.56
MSDCNN	29.7641	0.8534	0.0685	3.5018	0.9562	0.7915	48.39	1.01
Our	**30.1202**	**0.8659**	**0.0626**	**3.3609**	**0.9601**	**0.8092**	33.10	8.93

**Table 5:** Quantitative evaluation on the Pavia dataset (bold indicates the best results).

Methods	RMSE	PSNR	ERGAS	SAM	TIME(MS)
SSFCNN	7.9904	30.0794	4.9884	10.4791	47.18
ConSSFCNN	4.2634	35.5356	4.7384	4.5413	43.78
SSRNET	3.3973	37.5082	3.8721	3.7956	38.47
MSDCNN	4.2402	35.583	4.7711	5.3778	113.93
TFNET	3.4579	37.3545	3.8706	4.4395	168.31
ResTFNet	3.2405	37.9185	3.6348	4.0871	141.48
Our	**2.5813**	**39.8939**	**2.9413**	**3.5523**	**34.01**

**Table 6:** Quantitative evaluation on the Botswana dataset (bold indicates the best results).

Methods	RMSE	PSNR	ERGAS	SAM	TIME(MS)
SSFCNN	1.2083	29.5827	12.1663	5.7059	130.24
ConSSFCNN	1.7316	26.4576	18.0627	8.0279	91.67
SSRNET	0.7067	34.2422	14.4973	3.4285	94.98
MSDCNN	0.5411	36.5612	2.6302	2.5187	197.18
TFNET	0.4170	38.8247	2.1322	1.9714	166.36
ResTFNet	0.3773	39.6921	1.9042	1.7592	178.96
Our	**0.3402**	**40.5929**	**1.3636**	**1.5545**	**32.92**

## Data Availability

Data sharing is not applicable to this article.

## Abbreviations

The following abbreviations are used in this manuscript:

MS	Multi-spectral
PAN	Panchromatic
CS	Component substitution
MRA	Multiresolution analysis
CNN	Convolutional neural network
HSI	Hyperspectral image
GAN	Generative adversation network
SR	Super resolution
HRMS	High-resolution MS image
OFFM	Optimized feature fusion module
PReLU	Parametric rectified linear unit
PSNR	Peak signal-to-noise ratio
SSIM	Structural similarity
SAM	Spectral angle mapper
ERGAS	Relative dimensionless global error in synthesis
SCC	Spatial correlation coefficient
Q	Quality
PCA	Principal component analysis
IHS	Intensity-based saturation
RCAN	Very deep residual channel attention networks
CBAM	Convolutional block attention module
MGH	MTF-GLP with high-pass modulation
